# Towards improved balance exercise recommendations for older adults: integrating balance intensity into training dosage

**DOI:** 10.1186/s11556-026-00417-x

**Published:** 2026-06-09

**Authors:** Melanie Farlie, Kathryn Sibley, Gregory Brusola, Deborah Espy, Laura Jolliffe, Marissa Lyon, Caitlin McArthur, Yoshiro Okubo, Danielle Bouchard, Terry Haines

**Affiliations:** 1 School of Primary and Allied Health Care, Monash University, Melbourne, Australia; 2 Department of Community Health Sciences, Rady Faculty of Health Sciences, University of Manitoba, Winnipeg, Canada; 3 Department of Physical Therapy and Rehabilitation Sciences, School of Health Professions, The University of Texas Medical Branch at Galveston, Galveston, USA; 4 College of Health and Center for Human-Machine Systems, Cleveland State University, Cleveland, USA; 5 Ngarnga Centre, Peninsula Health, Frankston, Australia; 6 Department of Physical Therapy, University of New England, Portland, USA; 7 School of Physiotherapy, Dalhousie University, Halifax, Canada; 8 Neuroscience Research Australia, Sydney, Australia; 9 School of Population Health, UNSW Sydney, Sydney, Australia; 10 Faculty of Kinesiology, University of New Brunswick, Fredericton, Canada

**Keywords:** Postural balance, Exercise therapy, Exercise intensity, Accidental falls, Physical functional performance, Older adult

## Core content of this comment article

Balance exercise intensity is an under-researched dosage variable compared to strength and aerobic training, limiting evidence-based exercise guidance for older adults. A standardised balance intensity metric and feasible sampling approaches could accelerate research to identify the optimal intensity of balance exercise informing, clinical recommendations for preventing falls and promoting functional independence in older adults. Establishing this optimal level of balance intensity involves defining how balance effort is best described and measured, so that exercise programs can be tailored more precisely to target what matters most to older adult exercisers. This article provides commentary on various ways to conceptualise and measure exercise intensity, and discusses the strengths, limitations, and assumptions of each approach.

## Background

The capacity of people to adapt to physiological, social and environmental changes as they age is linked to positive ageing and wellness [1]. Recent global recommendations confirm that balance exercises should be tailored to the individual, and there is high-certainty evidence that what researchers consider to be challenging or high intensity balance exercises effectively improve balance performance and reduce falls [2].

“Balance intensity” is distinct from balance performance metrics (e.g., sway magnitude, reaction time, limits of stability) [3]. Balance intensity will also vary between individuals within task, for example, two individuals with different balance capacities will experience different balance intensities when performing the same task. Balance performance metrics and task difficulty hierarchies have been the mainstay of outcome measurement and balance exercise prescription in the field to date but do not capture the construct of balance intensity. Measures of balance intensity, such as the Balance Intensity Scale (BIS) [4] and Rating of Perceived Stability (RPS) [5], have recently been developed and validated. In particular, the BIS represents a proof-of-concept that the construct of balance intensity can be both self-perceived and rated directly by a trained observer. This marks an important advance in balance exercise research.

However, the present article does not seek to introduce, refine, or compare specific measurement instruments. Instead, we will use the BIS as an illustrative example that motivates a more fundamental question: how should balance intensity be conceptualised, and which dimensions of balance challenge are most relevant to this construct, independent of how they are ultimately measured? In this commentary, we will discuss multiple ways balance intensity measurement can be conceptualised during activity, and critique methodological approaches for collecting balance intensity data. A critical first step to the design of dose response studies that can inform and improve balance exercise recommendations for older adults.

Balance exercise primarily affects the neuromuscular, vestibular, oculomotor, and cognitive systems [6, 7]. Research on the impact of varying intensities of balance training lags decades behind studies on strength and cardiorespiratory training, limiting the evidence base for guidance on balance exercise for older adults. For example, the 1-repetition maximum (1RM) method for determining strength-training intensity was established in the 1940s [8]. Subsequent research identified that undertaking progressive resistance training at 80% of 1RM maximised strength gains, demonstrating the value of incorporating the intensity construct into exercise prescription [9].

There is a need to identify and apply the optimal intensity of balance training to maximise outcomes, in ways that are feasible for research and clinical applications during either structured training or daily activities. A complicating factor in this measurement is that a person’s balance performance is inherently dynamic and can change rapidly within a given task due to practice effects. Consider a person balancing on a beam: the intensity may be high at task initiation and then decrease to a more moderate level as they adapt to the task requirements over time, diminishing the difficulty of this task for the individual such that it is no longer a challenge. The task’s challenge relative to the individual’s balance capacity at a given point in time is the critical factor in determining the optimal dose of balance exercise training. This means that balance tasks in dose-response studies, as well as within individual balance training sessions, will need to be tailored to maintain a consistent intensity across participants and within sessions. Arising from this, there are two key issues to consider in the design of trials to examine the optimal dosage of balance exercise: when to collect data points (i.e., intensity ratings) and how to combine them for analysis. This paper will discuss approaches to address these issues and propose new metrics to communicate the intensity of balance-challenging activities.

## Main text

### Conceptual approaches to balance intensity during an activity

**Fig. 1:**
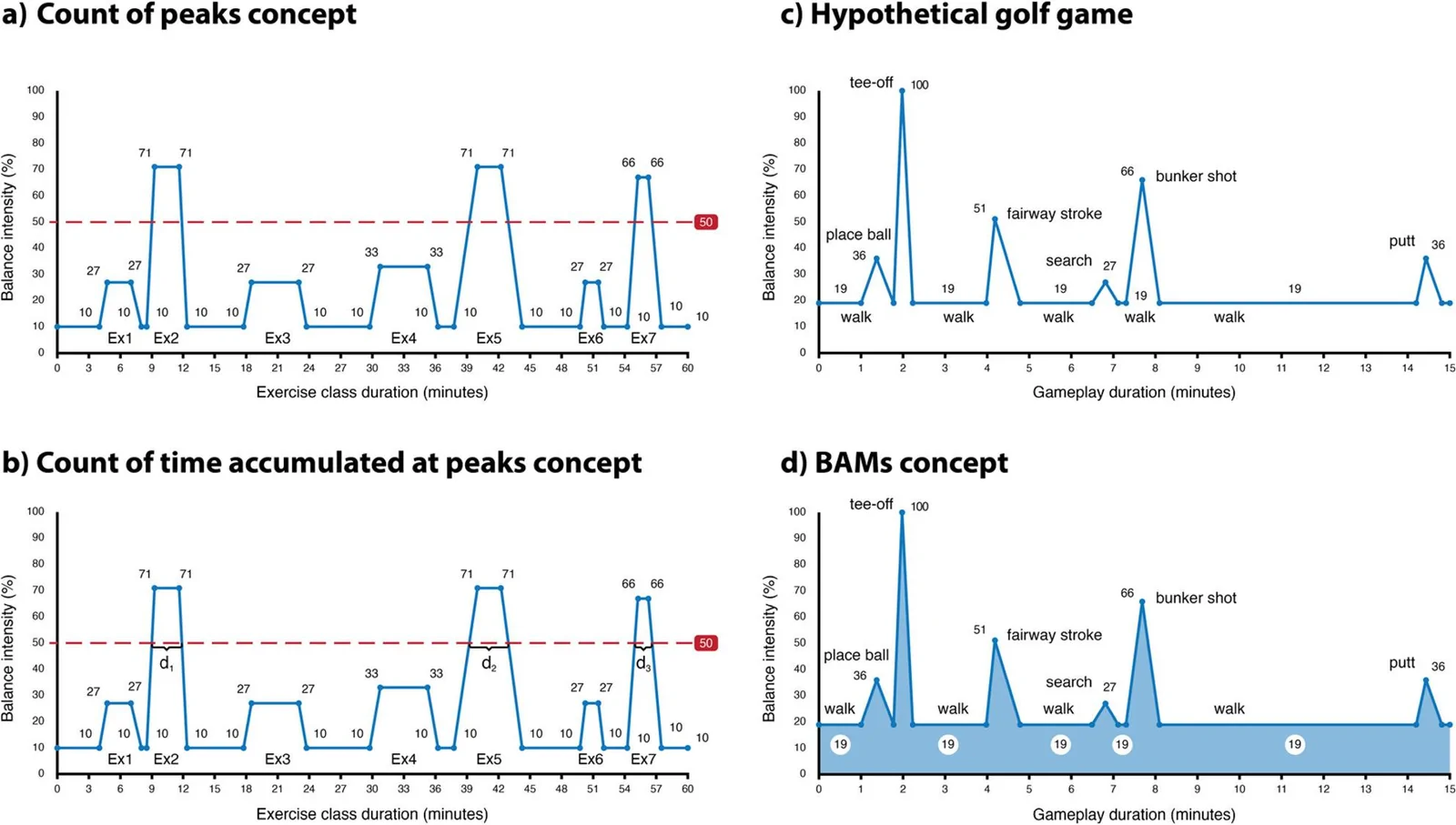
**a** Count of peaks over a threshold across the duration of a balance exercise circuit class. **b** Time spent over a threshold in same class. **c** Components represented in 15 minutes of a hypothetical golf game with associated fictional balance intensity percentages. **d** Balance-Intensity-Adjusted Minutes (BAMs) for golf game

We propose four potential approaches to conceptualising the measurement of balance intensity during an activity. For these conceptualisations, we deliberately move away from questions of how balance intensity is measured and instead focus on identifying the dimensions of balance challenge that are theoretically relevant to the construct. It is important to acknowledge that our conceptualisations of balance intensity measurement are theoretical, and each has limitations. To help contextualise each theoretical conceptualisation we draw parallels with similar measurements of other constructs. Our proposed conceptualisations, presented in Table 1, demonstrate how time-series balance intensity ratings can be used to assess the intensity of the experienced activity. Each conceptualisation is illustrated in Fig. 1, based on hypothetically generated data (see the supplementary file for the workbook). Illustration examples are inclusive of both structured balance activities (e.g., balance exercises delivered in circuit training) and daily activities (e.g., leisure activities that may confer balance benefits). With our conceptual focus in mind, the following section outlines distinct ways in which balance intensity can be characterised over time, independent of any measurement approach. Where numeric examples are required, percentage intensity values and time in minutes are shown for illustrative clarity, without implying reliance on a single measurement instrument, a definitive exercise duration, or a distinction between total exercise time and time spent at a given intensity level.

**Table 1:** Four proposed ways to conceptualise balance intensity data, drawing from examples used in other types of exercise, possible applications to the balance exercise context and potential limitations of each conceptual approach

**Conceptualisation 1 - Counting the number of peaks over a threshold**: counts the number of times the activity pushes participants to the limit of their balance capabilities. Figure 1a illustrates this using a hypothetical exercise class; the participant has completed seven exercises across the class (Ex1…Ex7). Percentage balance intensity during the class (x-axis values on the line chart) is recorded for each exercise and rest period. The (purely illustrative and entirely arbitrary) threshold for counting a peak has been set at 50%. In this class, the participant completed three circuit exercises at intensities greater than 50%, corresponding to 3 peaks in the hour. (Worksheet in supplementary material)		
**Example from another context**: An exercise session for a cyclist seeking to enhance their sprint capacity at the end of a race may be trained by lifting their intensity (peaks of intensity) for several short time intervals, achieving their maximum watts output (the threshold) on five occasions (the count) during their 30-minute session (the period) [10].	**Potential application to balance exercise**: May fit well with perturbation-based training, also referred to as reactive balance training [7]. Perturbation-based balance training involves unexpected, large perturbations, such as exposure to slip- or trip-walkways or waist pulls, to elicit reactive balance responses. Peaks to count may be indicated by the need for compensatory stepping or a harness-assisted recovery (peak) [11].	**Limitations of the approach**: Insensitive to what happens between the peaks, and the duration of the peak being exceeded. Also, it is insensitive to the actual intensity achieved.
**Conceptualisation 2 - Counting time accumulated at peaks**: measures time over and above an intensity or within a particular range. Figure 1b illustrates this in the same hypothetical exercise class, where the participant has completed seven exercises across the class (Ex1…Ex7). In this class, the participant completed three exercises on the circuit at a balance intensity above the (arbitrary) threshold of 50% for d1 (3 min), d2 (4 min), and d3 (2 min), for a total of 9/60 minutes above the threshold. (Worksheet in supplementary material)		
**Example from another exercise context**: Performing cardiorespiratory exercise above a particular Borg RPE rating for a minimum duration across a week. Gim and Choi [12] used this approach to investigate the impact of weekly exercise time on VO_2_ max and resting metabolic rate.	**Potential application to balance exercise**: It may be helpful if the outcome of interest is fall resistance [13]. The time spent performing very high intensity, falling or almost-falling tasks (peaks) has the most considerable training impact [14], with relatively less time spent on tasks, because fewer events, less often, are needed to boost and maintain the ability to resist falls [15].	**Limitations of the approach**: Insensitive to the degree to which the intensity threshold is exceeded within each peak, what happens below the threshold level and how far above the threshold is reached. These fluctuations may or may not be important, depending on the benefits of sub-maximal efforts. Fear of falling and environmental constraints may preclude the use of this approach.
**Conceptualisation 3 - Balance-Intensity-Adjusted Minutes (BAMs)**: a new metric we propose. This metric is obtained by repeatedly mapping balance intensity measures over time and then calculating the area under the intensity–time curve, capturing the combined influence of intensity level and duration. This approach summarises overall balance demand and does not correspond to the time spent at maximal intensity. Figure 1c illustrates a hypothetical mapping of the balance demands of a game of golf to help explain this potential application of this conceptualisation. Game components represented for 15 min of the hypothetical golf game, along with associated fictional balance intensity percentages (x-axis values on the line chart). Elements such as the walking pose minimal balance challenge (19%), whereas components such as the tee-shot pose a maximal balance challenge (100%). Using the fictional golf game illustrated in Fig. 1d, the area under the curve would equate to 3.39 BAMs in this passage of play. Using this conceptualisation, that means the 15-minute passage of play corresponds to 3.39 min of balance exercise at 100% intensity (Worksheet in Supplementary material).		
**Example from another context**: Has some intuitive appeal and a common approach for managing intensity data. For example, the intensity of cardiorespiratory exercise performed by a person riding an exercise bike at a gym can be quantified by integrating Watts over time to obtain a summary measure per hour of exercise (Kilowatt-Hours). Calculates the area under the curve of serial intensity measurements over time.	**Potential application to balance exercise**: Consider a game of golf in broad terms, where players experience varying levels of challenge throughout gameplay (Fig. 1c). Different balance challenges encountered during a round of golf relate to the various elements of the game, for example, the golf swing compared with walking between holes. The area under the curve of the different balance intensities experienced throughout the game could be used to calculate the BAMs.	**Limitations of the approach**: Treats a one-hour exercise program that challenges a person’s balance at 10% intensity and a six-minute program at 100% intensity equally.
**Conceptualisation 4 - BAMs over a threshold (BAMOTs)**: all BAMs below a certain threshold intensity are excluded from the end calculation. Figure 2a illustrates this, again using the fictional golf game. Using a (purely illustrative and entirely arbitrary) threshold of 25% intensity level, in this instance, would equate to 0.86 BAMOTs in this passage of play. Using this conceptualisation, that means a 15-minute passage of play corresponds to 0.86 min of balance exercise at 100% intensity. (Worksheet in Supplementary material).		
**Example from another context**: Fewer illustrated examples are available for this measurement approach. Those available, such as the quality-adjusted passing student educated (QAPSE) measure, are highly contextualised [16].	**Potential application to balance exercise**: In this approach, a minimum threshold is set to define a meaningful intensity level; all activity above this threshold is included in the area-under-the-curve calculation. For example, in a group exercise program designed to improve participants’ performance on more challenging exercises, there may be periods during the session when they are walking, standing, or seated that are not relevant to this context.	**Limitations of the approach**: The cost-effectiveness of this data collection approach may appeal to researchers with limited resources or clinicians with limited time. However, it is predicated on the unproven hypothesis that intensities below a particular threshold provide no clinical benefit. But this hypothesis is yet to be proven.

### Methodological approaches to balance intensity data collection for an activity

**Fig. 2:**
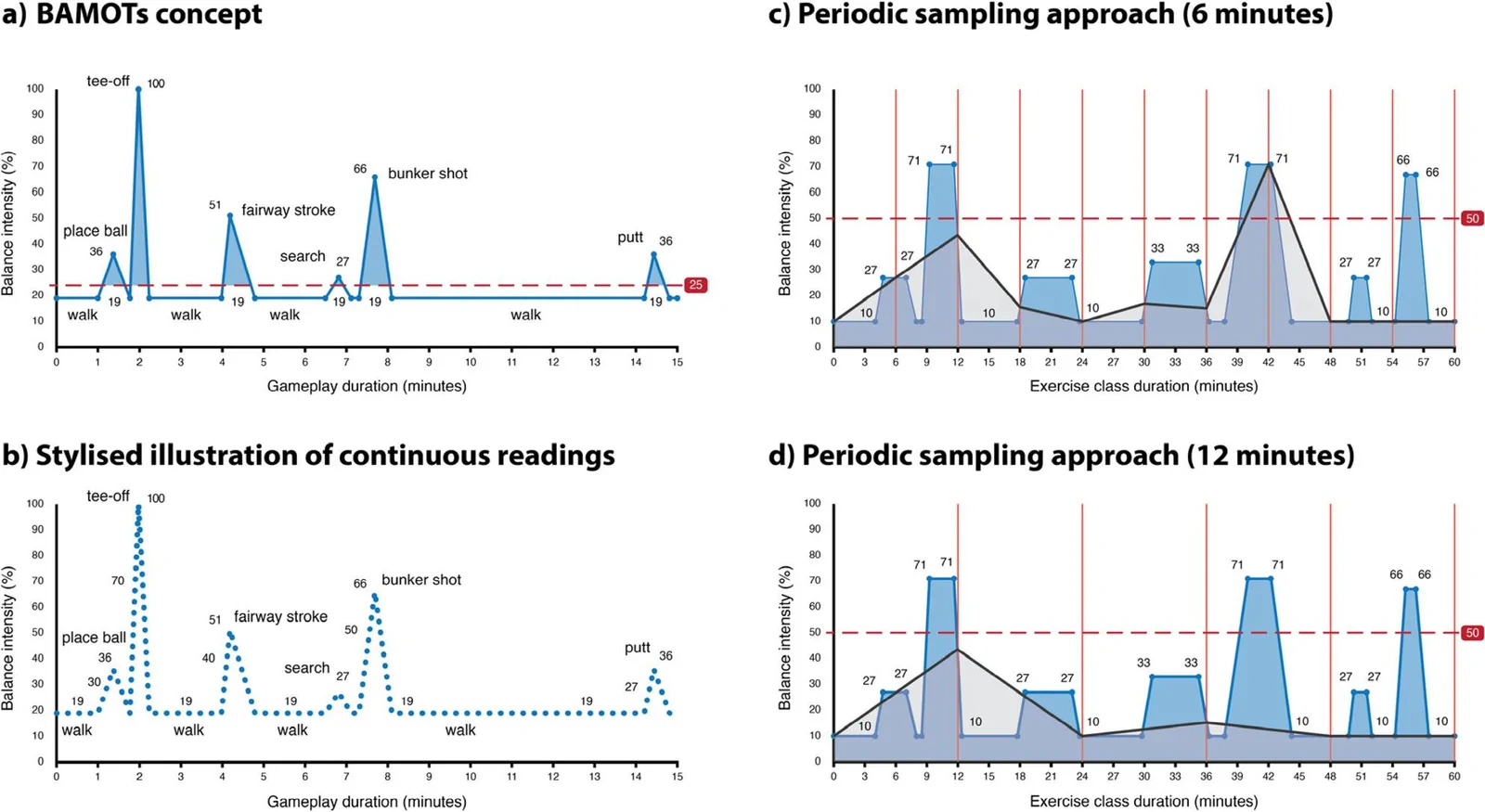
**a** BAMs over a threshold (BAMOTs) during the golf game. **b** Continuous sampling during a hypothetical game of golf. Not all data points are labelled. **c** Periodic sampling every six minutes (vertical red lines) in the balance exercise circuit class. **d** Periodic sampling every 12 minutes (vertical red lines)

The four proposed conceptualisations require repeated assessments of balance intensity during an activity. There are several ways these data could be collected, each with its potential ramifications for data accuracy and feasibility. We consider five approaches to collecting balance intensity data during an activity. It is unclear which of these options represents the best approach for clinicians and researchers interested in describing the intensity of the balance challenge in the programs and activities they prescribe, as well as in individuals engaged in physical activity. However, in examining these options, we hope to encourage broader exploration and dialogue within the balance exercise community. We explore these approaches in Table 2 and again provide analogous examples of their application in other contexts. The underlying assumption we apply in this paper is that for an individual engaged in physical activity, the intensity of the balance challenge varies continuously. Consider the earlier example of playing golf, which involves a series of activities that range in balance intensity, such as walking from green to green, bending to place the ball, swinging, and retrieving the ball. We have illustrated this hypothetical game in Fig. 2b. Currently, the logistics and costs of continuous sampling of balance intensity using conventional data-collection methods are not viable. This will remain the case until options such as artificial intelligence-based processing of video-captured data are validated to perform such a function. Therefore, in Table 2, we propose a range of alternate approaches that can be used where this capability is not yet available in research and clinical contexts.

**Table 2:** Five methodological approaches to the capture of balance intensity data, feasibility in the balance exercise context and potential limitations of each approach

**Methodological approach 1 - Continuous sampling**: Continuous, uninterrupted stream of collected data. Measure of balance intensity of all components of a task or series of tasks. A comprehensive indication of balance intensity. Figure 2b illustrates the continuous sampling process during a hypothetical game of golf, in which moment-by-moment intensity readings are recorded.	
**Feasibility considerations**: Determining the instantaneous intensity of a balance challenge can be difficult when considering the speed of specific components of the activity, such as the golf swing. This may be accomplished using recorded footage that can be examined for visual indicators of balance challenge [4] frame by frame, but it continues to impose resource demands that may be feasible only in a research trial.	**Limitations of the approach**: The time and resources required to do this and, therefore, the practicality of its implementation. A person’s self-rating of the moment-to-moment intensity of the balance challenge would likely confound the task, rendering it a dual-task. For observer or self-ratings, the method is labour-intensive and potentially impractical.
**Methodological approach 2 - Periodic sampling - set or random intervals**: involves data collection at fixed intervals, with equal time intervals between data collection points. Therefore, the critical issues are the frequency of data collection and the consequences of this interval choice for data accuracy and feasibility. Figure 2c returns to the hypothetical 60-minute balance exercise class with seven balance exercise stations. Sampling every six minutes yields intensity readings used to calculate the area under the curve (foreground shading), which, in this example, corresponds to 13.62 BAMs or 8.52 BAMOTs when using the arbitrary threshold of balance intensity greater than 25%. The count of peaks above 50% would be 1, and the duration above 50% is the duration of the one observation at minute 42. Figure 2d shows a sampling interval of 12 min for the same class; this corresponds to 11.58 BAMs and 5.28 BAMOTs. The count of peaks over 50% would be zero, and the duration of time of peaks over 50% is zero. Finally, Fig. 3a shows that random sampling yields intensity readings of 20.35 BAMs. This corresponds to 20.35 min of exercise at 100% intensity, or 15.76 BAMOTs, when using the arbitrary 25% threshold. This equates to 20.35 min of exercise at 100% intensity. The count of peaks > 50% is 3, and the duration over 50/100 is the duration of the three observations at minute 10.8, 39.6 and 42.3. (Worksheets in Supplementary material).	
**Feasibility considerations**: More resource-efficient as observations are spaced apart. This may increase the feasibility and accuracy of data collection. Random sampling may further mitigate the systematic biases associated with set-interval data collection methods. The benefits of the random interval approach are similar to those of clinical trialists who prefer random allocation to alternative allocation methods to mitigate systematic bias.	**Limitations of the approach**: A risk with the set and random periodic interval sampling methods is that short-duration, high-intensity measures may be missed because they occur between the cross-sectional samples. As demonstrated in the fictional balance exercise class (Figs. 2c, d, c and d and 3a), depending on the sampling method used, the balance intensity-adjusted minutes ranged from 11.58 to 20.34 BAMs, and the peak count ranged from 0 to 3. Systematic bias can be mitigated using random interval sampling, but all period sampling approaches risk missing important data.
**Methodological approach 3 - Purposive sampling**: This approach requires defining discrete task components and specifying their start and end times, for example, rating the intensity of the drive from the tee in a game of 18 holes of golf. If the intensity of the balance challenge during the first tee shot and the duration of time taken to complete the shot were observed, the intensity and duration of this shot could then be applied to the other 17 tee shots in the game. This process could be repeated for all different parts, such as walking between shots, fairway shots, putting and retrieving the ball from each hole. Figure 3b shows where elements (e.g., placing ball - yellow, tee off - green, etc.) are measured for the whole duration of an activity or a sample of an activity and extrapolated to the entire duration. See the worksheet in Supplementary material for the BAMs, BAMOTs, count and duration of peaks for each element for these 15 min of gameplay.	
**Feasibility considerations**: If purposively sampled data are combined with the duration of each component, they can be used to calculate BAMs or BAMOTs and to count peaks or durations. Each task component gets sampled and represented, avoiding the risk that meaningful activities will be missed. This method has increased feasibility for use in research and clinical contexts, as a small sample of data can be paired with self-report data and used to estimate intensity over durations beyond the observation period.	**Limitations of the approach**: Incorrect assumptions about the homogeneity of activity components or durations would reduce data accuracy. For example, the tee shot on the first hole might take considerably longer or be more challenging than subsequent tee shots and therefore over-inflate the intensity of the game when applied to the other 17 tee shots in the game.
**Methodological approach 4 - Retrospective summative**: This approach involves summarising an entire task or period of activity with a single ‘average’ measure, nominated by either the person completing the activity or an external observer. The retrospective summative method, applied in the context of an exerciser self-rating the average balance intensity of a balance exercise class, is illustrated in Fig. 3c. The rating is provided at the end of the exercise class, as indicated by the star. The heavy dotted line illustrates a hypothetical representation of the recall of an exerciser who is asked to rate using balance intensity, ‘on average, how hard did you need to work to maintain your balance in today’s class?’ with the response equating to 40%. The balance intensity (calculated under the light dotted line) would equate to 24 BAMs and zero BAMOTs after setting the BAMOT threshold at greater than 25%. The balance intensity would have zero peaks and zero duration above the peak threshold if using a peak-count or duration threshold of 50% or greater (Worksheet in Supplementary material).	
**Feasibility considerations**: Separates the task’s rating from its performance. This may mitigate potential confounding introduced by including rating within the activity performance period. Methodologically expedient, which may increase the feasibility. This approach is analogous to using a Borg rating [17, 18] to quantify the perceived intensity of a 1-hour circuit-training session.	**Limitations of the approach**: Ambiguity about the frame of reference may lead to confusion about what is being measured and complicate the interpretation of retrospective summative scores. Retrospective recall likely varies with the intensity and duration of the activity, and with the time since completion. May be subject to recall bias or confusion about the reference activities. Different levels of balance intensity may be perceived depending on whether a person recalls the intensity at the start, at the point where they found the activity most challenging, or at the end, when they may have felt more confident.
**Methodological approach 5 - Retrospective continuous**: An observer or exerciser performing a self-rating would be asked to construct something akin to a timeline or journey map, in which the intensity of the balance challenge of an immediately past activity would be recalled, along with the associated duration of each intensity experienced or observed. Figure 3d shows the intensity of seven balance exercises in a 60-minute balance exercise class. The intensity recalled is represented by the red dashed line. These data can be used to calculate BAMs, BAMOTs, and peak counts. Adding video replays to aid recall of each exercise’s duration may be necessary to improve recall of peak duration.	
**Feasibility considerations**: Reducing the confounding of the continuous sampling method. It may be advantageous for mitigating recall bias, as the specific activities rated during the reference period are defined. Access to playback, for example, viewing a video recording of the activity, could enhance recall. For example, in continuous sampling, a person may rate a portion of an activity at high intensity but then experience another portion at a higher intensity, which may alter their perception of the earlier activity’s intensity.	**Limitations of the approach**: May be subject to recall bias and response shift, where retrospective ratings may be more or less favourable than real-time ratings. In retrospective data capture, later experiences can influence the interpretation of earlier experiences [19, 20].

### Setting the agenda for balance-intensity research in older adults

There is already strong evidence that falls can be prevented through balance exercise programs. The World Falls Guidelines recommend that balance exercise needs to be challenging and progress in intensity [2]. Despite this, fall-related hospital admissions continue to rise globally [21], and there is evidence that balance training programs are being delivered at low intensities [22, 23]. Clinicians need to know how to increase and effectively progress the intensity of the balance training they prescribe, and what intensity targets they need to reach to best address this problem.

**Fig. 3:**
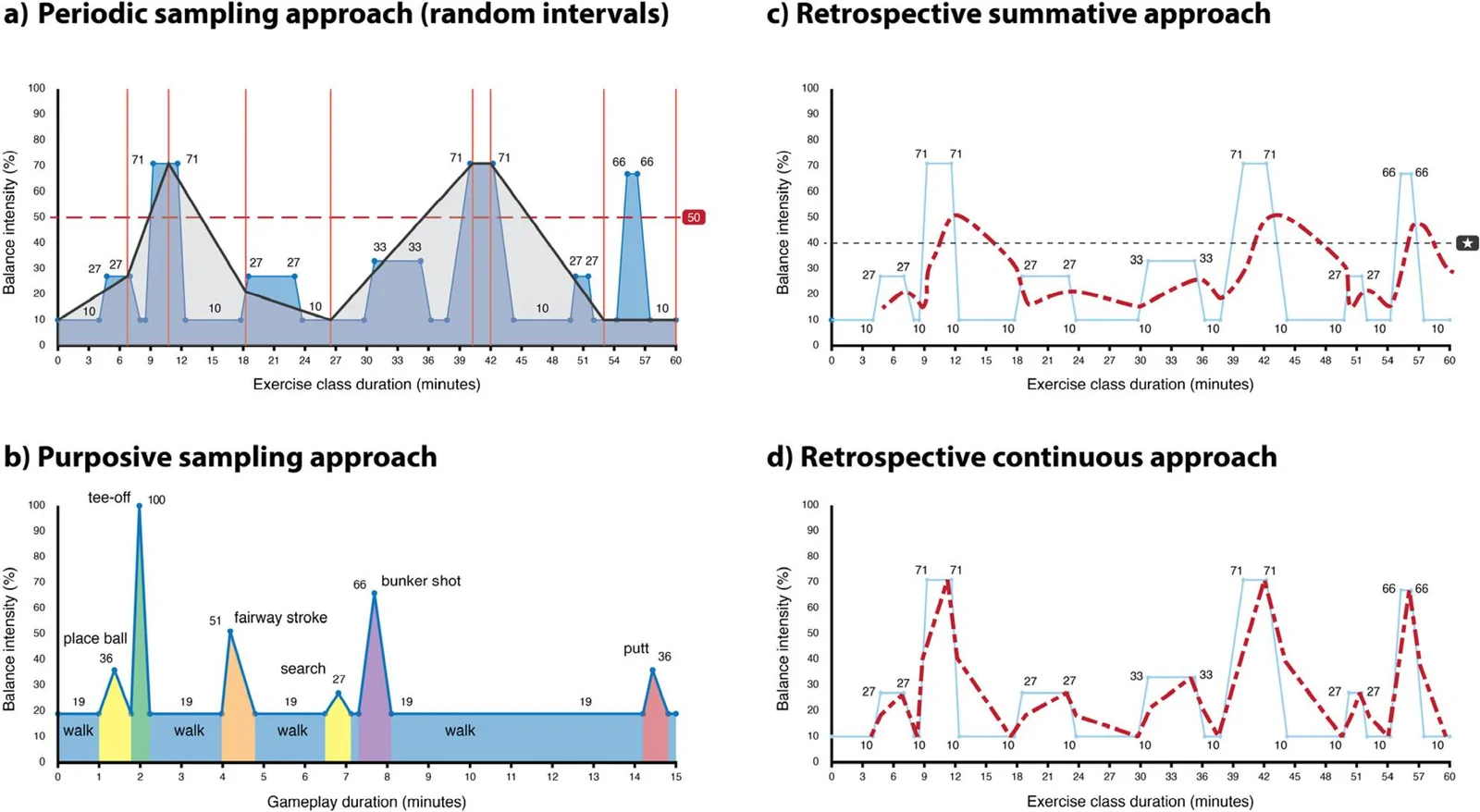
**a** Sampling at random intervals (vertical red lines). **b** Purposive sampling approach. **c** Retrospective summative balance intensity rating (star symbol). **d** Retrospective continuous observer recall

Ideally, research characterising the relative benefits of different intensities of training programs, as conceptualised across the different means we have described, will be published. It may be that there is little dose-response relationship with outcomes across some conceptualisations of intensity. These conceptualisations could be abandoned in favour of others where a stronger relationship exists. Once a conceptualisation is identified with the strongest dose-response relationship, then identifying the optimal intensity prescription within this conceptualisation for different patient groups and contexts can proceed.

Research examining the balance intensity challenge posed by leisure-based and other daily activities would also be of value, as some patients are reluctant to participate in formal balance training programs [24], yet may be willing to engage in everyday activities that pose a sufficient balance challenge to yield a training effect.

## Conclusion

Compared with resistance and cardiorespiratory exercise, the field of balance exercise is decades behind in understanding the dose-response relationship. This lag is partly due to the complexity of the variables and current technical limitations. We have proposed several conceptualisations of balance intensity measurement and data-collection methods that could guide the design of coordinated dose-response trials. While there are philosophical dilemmas, such as the paradox of studying a dose-response relationship that may influence the approach used, we believe these complex challenges are not insurmountable. The potential population health benefits far outweigh the effort required to establish definitive evidence on the optimal intensity of balance-challenging physical activity to achieve meaningful improvements in health and well-being in our community.

## Supplementary Information

Supplementary Material 1.

## Data Availability

No datasets were generated or analysed during the current study.
