# Supplementary material for: Towards improved balance exercise recommendations for older adults: integrating balance intensity into training dosage
Source: Eur Rev Aging Phys Act. 2026 Jun 9;23:20. doi: 10.1186/s11556-026-00417-x (PMC13248376; doi:10.1186/s11556-026-00417-x)
Supplement: Supplementary file 1 — Supplementary Material 1. [file 11556_2026_417_MOESM1_ESM.pdf]

Glossary

AUC – Area under the curve.

Area under the curve is a way of summarising overall load or challenge by combining how intense something is with how long it lasts.

BAMs – Balance intensity Adjusted Minutes.

Instead of only counting the time spent at the highest intensity, this metric represents an area under the curve approach to consider all intensity levels across an activity. Higher intensities contribute more to the total, while lower intensities contribute less, but both still matter. When plotted over time, the area under the intensity curve represents the total accumulated challenge experienced during the activity.

BAMOTs – Balance intensity Adjusted Minutes Over a Threshold

This metric describes the total amount of challenge experienced only when intensity exceeds a specified level. Rather than considering all intensities, this approach focuses on the portion of activity where the intensity rises above a chosen threshold (for example, moderate or high intensity). Both how far above the threshold the intensity is and how long it remains above that level contribute to the total. This measure is useful for capturing exposure to higher-intensity demands, rather than overall accumulated challenge.

Worksheets

Worksheet for Figure 1d, 2a

AUC = sum of (minute-time elapsed)x(balance intensity score/100)

Minute	Balance Intensity Scale score	Time elapsed	Percentage intensity	AUC	AUC when BIS>25/100
0.001	19	0.001	0.19	0.00019	0
1	19	0.999	0.19	0.18981	0
1.4	36	0.4	0.36	0.144	0.144
1.8	19	0.4	0.19	0.076	0
2	100	0.2	1	0.2	0.2
2.2	19	0.2	0.19	0.038	0
4	19	1.8	0.19	0.342	0
4.2	51	0.2	0.51	0.102	0.102
4.8	19	0.6	0.19	0.114	0
6.5	19	1.7	0.19	0.323	0
6.8	27	0.3	0.27	0.081	0.081
7.1	19	0.3	0.19	0.057	0
7.3	19	0.2	0.19	0.038	0
7.7	66	0.4	0.66	0.264	0.264
8.1	19	0.4	0.19	0.076	0
14.2	19	6.1	0.19	1.159	0
14.4	36	0.2	0.36	0.072	0.072
14.8	19	0.4	0.19	0.076	0
15	19	0.2	0.19	0.038	0
				**3.39 BAMs**	**0.863 BAMOTs**

Worksheet for Figure 2c, 2d and 3a: continuous sampling calculations (same background continuous sampling calculation for all three graphs)
AUC = sum of (minute-time elapsed)x(balance intensity score/100)

Minute	Balance Intensity Scale score	Time elapsed	Percentage intensity	AUC	AUC when BIS>25/100
0.01	10	0.01	0.1	0.001	0
4	10	3.99	0.1	0.399	0
5	27	1	0.27	0.27	0.27
7	27	2	0.27	0.54	0.54
8	10	1	0.1	0.1	0
8.5	10	0.5	0.1	0.05	0
9	71	0.5	0.71	0.355	0.355
11.5	71	2.5	0.71	1.775	1.775
12.5	10	1	0.1	0.1	0
18	10	5.5	0.1	0.55	0
19	27	1	0.27	0.27	0.27
22.5	27	3.5	0.27	0.945	0.945
23.8	10	1.3	0.1	0.13	0
30	10	6.2	0.1	0.62	0
31	33	1	0.33	0.33	0.33
35	33	4	0.33	1.32	1.32
36	10	1	0.1	0.1	0
38	10	2	0.1	0.2	0
40	71	2	0.71	1.42	1.42
42	71	2	0.71	1.42	1.42
44	10	2	0.1	0.2	0
50	10	6	0.1	0.6	0
50.5	27	0.5	0.27	0.135	0.135
51.5	27	1	0.27	0.27	0.27
52	10	0.5	0.1	0.05	0
54	10	2	0.1	0.2	0
55	66	1	0.66	0.66	0.66
56	66	1	0.66	0.66	0.66
57	10	1	0.1	0.1	0
60	10	3	0.1	0.3	0
				**13.77 BAMs**	**10.37 BAMOTs**

Worksheet for Figure 2c: sampling at 6-minute interval calculations
AUC = sum of (minute-time elapsed)x(balance intensity score/100)

Minute	Balance Intensity Scale score	Time elapsed	Percentage intensity	AUC	AUC when BIS>25/100
0	10	0.01	0.1	0.001	0
6	27	6	0.27	1.62	1.62
12	44	6	0.44	2.64	2.64
18	15	6	0.15	0.9	0
24	10	6	0.1	0.6	0
30	16	6	0.16	0.96	0

36	14	6	0.14	0.84	0
42	71	6	0.71	4.26	4.26
48	10	6	0.1	0.6	0
54	10	6	0.1	0.6	0
60	10	6	0.1	0.6	0
				**13.621 BAMs**	**8.52 BAMOTs**

Worksheet for Figure 2d: sampling at 12-minute interval calculations
AUC = sum of (minute-time elapsed)x(balance intensity score/100)

Minute	Balance Intensity Scale score	Time elapsed	Percentage intensity	AUC	AUC when BIS>25/100
0	10	0.01	0.1	0.001	0
12	44	12	0.44	5.28	5.28
24	10	12	0.1	1.2	0
36	20	12	0.2	2.4	0
48	10	12	0.1	1.2	0
60	10	12	0.1	1.2	0
				**11.281 BAMs**	**5.28 BAMOTs**

Worksheet for Figure 3a: sampling at random interval calculations

Randomly generated intervals	Normalised to a total of 60 minutes	Minute	Balance Intensity Scale score	Time elapsed	Percentage intensity	AUC	AUC when BIS>25/100
0.0	0.0	0.0	10	0.0	0.10	0.000	0.000
6.8	6.8	6.8	27	6.8	0.27	1.849	1.849
3.9	3.9	10.8	71	3.9	0.71	2.771	2.771
8.1	8.2	18.9	25	8.2	0.25	2.048	0.000
7.6	7.7	26.6	10	7.7	0.10	0.770	0.000
12.8	12.9	39.6	71	12.9	0.71	9.163	9.163
2.8	2.8	42.3	71	2.8	0.71	1.981	1.981
10.4	10.5	52.8	10	10.5	0.10	1.048	0.000
7.1	7.2	60.0	10	7.2	0.10	0.718	0.000
59.4	60.0					**20.348 BAMs**	**15.764 BAMOTs**

Worksheet for 3b: Calculations of balance intensity adjusted minutes (BAMs), balance intensity adjusted minutes over a threshold (BAMOTs), counts and duration of peaks for the hypothetical golf game using the purposive sampling approach

Element	Time elapsed	BAMs	BAMOTs	Count of peaks	Duration of peaks
walking	13.3	2.527	0	0	0
searching	0.3	0.081	0.081	0	0

placing ball	0.6	0.216	0.216	0	0
fairway stroke	0.2	0.102	0.102	1	0.2
bunker shot	0.4	0.264	0.264	1	0.4
tee-off	0.2	0.2	0.2	1	0.2
TOTALS	15 minutes	3.39	0.863	3 peaks	0.8 minutes

Worksheet for Figure 3c: retrospective summative calculations

**minute**	**intensity (BIS-E)**	**Time elapsed**	**Percentage intensity**	**AUC**	**AUC when BIS>2/5**
60	2	60	0.4	24 BAMS	0 BAMOTs
